# Buoyant particulate strategy for few-to-single particle-based plasmonic enhanced nanosensors

**DOI:** 10.1038/s41467-020-16329-y

**Published:** 2020-05-25

**Authors:** Dongjie Zhang, Leqin Peng, Xinglong Shang, Wenxiu Zheng, Hongjun You, Teng Xu, Bo Ma, Bin Ren, Jixiang Fang

**Affiliations:** 1 Key Laboratory for Physical Electronics and Devices of the Ministry of Education, School of Electronic Science and Engineering, Faculty of Electronic and Information Engineering, Xi’an Jiaotong University, Xi’an, 710049 Shannxi P. R. China; 2 State Key Lab of Multiphase Flow in Power Engineering, Xi’an Jiaotong University, Xi’an, 710049 Shannxi P. R. China; 3 Single-Cell Center, CAS Key Laboratory of Biofuels and Shandong Key Laboratory of Energy Genetics, Qingdao Institute of BioEnergy and Bioprocess Technology, Chinese Academy of Sciences, Qingdao, 266101 Shandong P. R. China; 4 State Key Laboratory of Physical Chemistry of Solid Surfaces, Department of Chemistry, College of Chemistry and Chemical Engineering, Xiamen University, Xiamen, 361005 Fujiang P. R. China

**Keywords:** Raman spectroscopy, Nanophotonics and plasmonics, Nanosensors, Imaging and sensing

## Abstract

Detecting matter at a single-molecule level is the ultimate target in many branches of study. Nanosensors based on plasmonics have garnered significant interest owing to their ultrahigh sensitivity even at single-molecule level. However, currently, plasmonic-enhanced nanosensors have not achieved excellent performances in practical applications and their detection at femtomolar or attomolar concentrations remains highly challenging. Here we show a plasmonic sensing strategy, called buoyant plasmonic-particulate-based few-to-single particle-nanosensors. Large-sized floating particles combined with a slippery surface may prevent the coffee-ring effect and enhance the spatial enrichment capability of the analyte in plasmonic sensitive sites via the aggregation and lifting effect. Dimer and single particle-nanosensors demonstrate an enhanced surface-enhanced Raman spectroscopy (SERS) and a high fluorescence sensitivity with an enrichment factor up to an order of ∼10^4^ and the limit of detection of CV molecules down to femto- or attomolar levels. The current buoyant particulate strategy can be exploited in a wide range of plasmonic enhanced sensing applications for a cost-effective, simple, fast, flexible, and portable detection.

## Abstract (web summary)

Plasmonic-enhanced nanosensors are limited in practical applications, as it remains challenging to detect molecules at low concentrations. Here, the authors introduce a buoyant particulate strategy in order to enrich analytes in the plasmonic hot spots.

## Introduction

A plasmonic-enhanced nanosensing process involves complicated coupled three-body interactions among photons, molecules, and nanostructures^1–3^. In previous decades, fundamental issues regarding the interaction between light and nanostructures have been investigated intensively, and many types of plasmonic hot spots have been fabricated, e.g., nanogaps, nanotips, and nanopores, for enhanced surface-enhanced Raman spectroscopy (SERS) and fluorescence sensitivity^4–8^. However, SERS detection for many small cross-sections or weakly adsorbed molecules is still difficult owing to the deficiency of related investigations on the interaction between molecules and nanostructural surfaces. For example, in the commercial SERS protocol based on a colloidal aggregation route^9^, weakly adsorbed molecules cannot effectively adsorb onto a metallic surface during fast aggregation. Therefore, this natural defect makes them impossible to exhibit a remarkable sensitivity. In the form of SERS solid surface with precise nanopatterns^10^, dipping the SERS substrate in a solution containing analytes may yield a homogeneous molecule adsorption. However, the adsorption time (e.g., a few hours) is far beyond practical timescales. Instead, by drying a droplet containing analytes on a substrate, the molecule distribution on a substrate may encounter uniformity issue owing to the ubiquitous coffee-ring effect^11^, particularly in weakly adsorbed molecules. More importantly, relative to the large area of patterned surfaces, the hot spot sites (with areas of only a few square nanometers) possess only a small portion of the entire substrate, thereby resulting in poor molecular accessibility when only a few molecules are investigated. Currently, localizing analytes toward plasmonic hot spot sites with high-efficiency is paramount in improving the sensitivity of plasmonic-enhanced nanosensors.

The coffee-ring effect is a very common phenomenon, and its nature is that the capillary flow outward from the center of the drop carries dispersed particulates to the edge as evaporation proceeds^12^. In many detections based on plasmonic nanosensors, the formation of a coffee-ring may result in a completely uncontrolled distribution of both colloidal nanoparticles (NPs) and target molecules, resulting in deteriorated signal uniformity and sensitivity^13^.

Herein, we report a plasmonic-enhanced sensing strategy based on buoyant plasmonic particulates that are designed to thoroughly avoid the coffee-ring effect and guide target molecules into a spatially highly localized plasmonic hot spot region. The resulted dense-packed pattern, particularly for dimer or single particles, may significantly increase the sensitivity of plasmonic sensors with an enrichment factor of up to ∼10^4^. Combined with the developed procedure using a superhydrophobic surface to accurately sort single particles, the current buoyant particulate strategy is believed to be applicable to a wider range of sensing devices, such as fluorescent, Raman, and infrared spectroscopes for a cost-effective, simple, fast, flexible, and portable detection. We call this new technique as the buoyant particulate-based few-to-single particles-plasmonic nanosensor.

## Results

### Exact adsorption position of analyte

**Fig. 1:**
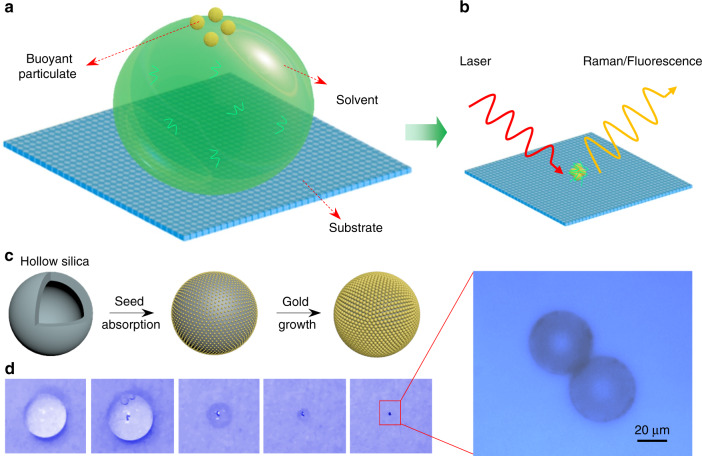
Schematic illustrations of the buoyant particulate strategy. **a** The proposed buoyant particulate protocol consisting of slippery surface, solvent, and floating particles. **b** After drying, the containing probe molecules and plasmonic floating particles may aggregate and condense together, thus enhance the sensitivity of plasmon-type nanosensors. **c** The schematic processes of synthesizing light-weight hollow silica-coated Au shell particles by a seed-mediated growth route. **d** The evaporation processes of suspended hollow silica-coated Au shell particles within a droplet and the final aggregated pattern. The scale bar represents 20 μm.

The strategy for the buoyant plasmonic particulates sensor is shown in Fig. 1a, b. Large-sized (30–100 μm), light-weight floatable particles, i.e., hollow SiO_2_ coated with Au nanoparticles, were synthesized by a seed-mediated growth route (Fig. 1c), which is a modified method described by Westcott et al., Shao et al., and Liu et al.^14–16^ (see Supplementary Figs. 1–3 for details). During the drying process, the buoyant particles may float on the top of the solvent, thereby significantly reducing their chances of being pinned on the slippery surface^17–19^ (Fig. 1d). The large size of the floating particles may increase the capillary force to drive these particles inward the droplet and aggregate as a dense-packed structure. In particular, at the final stage of evaporation, the landed particles on the substrate may serve as “post” to lift the droplet from the slippery surface, thereby enforcing the solvent and target molecule to dry in the vicinity of particle–particle junctions (Fig. 1d)^20^. Consequently, the obtained condensed pattern can significantly increase the sensitivity of the plasmonic nanosensors.

**Fig. 2:**
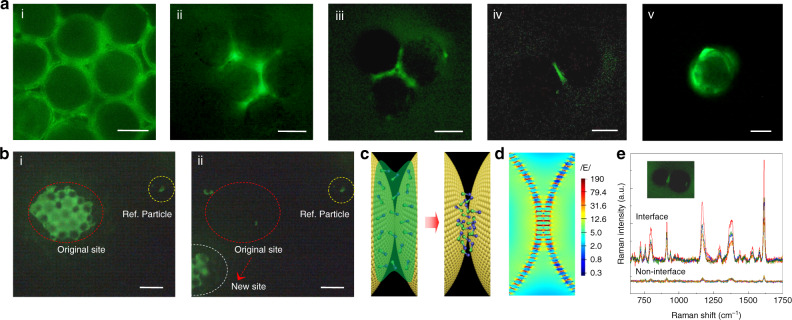
Enriching effect, lifting effect, and fluorescent characterizations. **a** Fluorescent images of floating particle aggregate with various CV concentrations. A strong fluorescent signal can be observed clearly in the vicinity of particle–particle interface. **b** Fluorescent images with CV concentration of 10^−8^ M. The red circle showing that after we moved the aggregate, the original site on the substrate does not display any fluorescent signal. **c** The schematic illustrations of the solvent ring finally adsorbs on the particle–particle interface using the present buoyant particulate protocol. **d** FDTD simulation of the plasmonic coupling sites of dimer buoyant particulates. **e** Raman spectra collected from the interface and non-interface regions for a dimer floating particles with CV concentration of 10^−9^ M. The insets are the fluorescent images of a dimer buoyant particulate, demonstrating a 10–20 times of signal difference between interface and non-interface regions. The scale bar in (**a**) is 20 μm and in (**b**) is 100 μm.

The most advantageous aspect of the current strategy is the aggregation effect of buoyant-particulates on a slippery surface (Fig. 1d and Supplementary Movie 1), which contributes to the enrichment of the solvent in the vicinity of the particle–particle interface. Consequently, target molecules that are highly localized toward plasmonic hot spot sites were achieved. Supplementary Figures 4 and 5 and Supplementary Movies 2 and 3 demonstrate clear aggregation and enrichment processes. To investigate the exact adsorbed position of the analyte, we firstly performed the fluorescent characterizations for the obtained aggregates using crystal violet (CV) molecules as a fluorescent probe. Figure 2a(i–v) shows the fluorescent images of buoyant particulates with different particle amounts dried on the slippery surface. A strong fluorescent signal can be observed clearly in the vicinity of the particle–particle interface for the configurations of more than two particulates. In the case of a single buoyant particulate, after the evaporation of the droplet, the analyte finally dries and adsorbs on/near the surface of the buoyant particulate according to the fluorescent image, as shown in Fig. 2a(v).

Next, we conducted an in-situ observation by moving the aggregate of landed particles (Supplementary Movie 4). It is interesting that after we moved the aggregate, the original site of the substrate did not display a network-shaped fluorescent signal (Fig. 2b(i and ii) and Supplementary Fig. 6). This phenomenon indicates that, after the drying of the solvent, the CV molecules do not adsorb on the substrate but in the vicinity of the particle–particle interface (Fig. 2a). To further verify this statement, two controlled experiments were conducted by only replacing the buoyant plasmonic particulates. Similar to the results shown in Fig. 2b, when we employed hollow bare SiO_2_ particles to enrich the CV molecules, the strong network-shaped fluorescent signal from the formed aggregate can still be observed (Supplementary Fig. 7). After moving the formed aggregate of hollow SiO_2_ particles, the fluorescent signal from the original site fully disappeared. However, when we evaporated a droplet containing only CV molecules on the slippery substrate, the fluorescent imaging of the CV molecules (Supplementary Fig. 8) was still detectable at concentrations down to the level of 10^−8^–10^−9^ M. Therefore, these comparison experiments revealed that, using the current buoyant plasmonic particulate strategy, after the drying of the droplet, the analytes mainly enrich and localize into the particle–particle interface, i.e., the hottest spot region.

The fluorescent characterizations of drying a droplet containing buoyant particulates support the fact that a lifting effect may occur at the final stage of evaporation. Once the buoyant particulates have landed on the substrate, these particles may serve as a “post” to enforce the solvent and analyte to interact with the post. Consequently, the solvent and probe molecules are driven and enriched into the particle aggregate. The occurrence of the lifting phenomenon requests the capillary force in the vicinity of the particle–particle interface should be larger than the gravitation of the solvent and the adhesion force between the solvent and the substrate. In fact, this is possible because the Teflon substrate used and the perfluorinated lubricant acting as a superhydrophobic and slippery surface contribute to a low surface energy, thereby reducing the adhesion force significantly.

Obviously, the aggregation and lifting effects are crucial in enforcing the solvent and analyte toward the particle–particle interface (Fig. 2c), i.e., additional plasmonic coupling site (Fig. 2d), which may significantly increase the sensitivity in many types of sensing. Figure 2e shows the Raman spectra for a dimer of buoyant particulate using the CV molecules as a probe. The interface regions display a remarkably enhanced signal intensity, e.g., 10–20 times, compared with that in non-interface regions.

### Droplet drying processes in buoyant particulate system

**Fig. 3:**
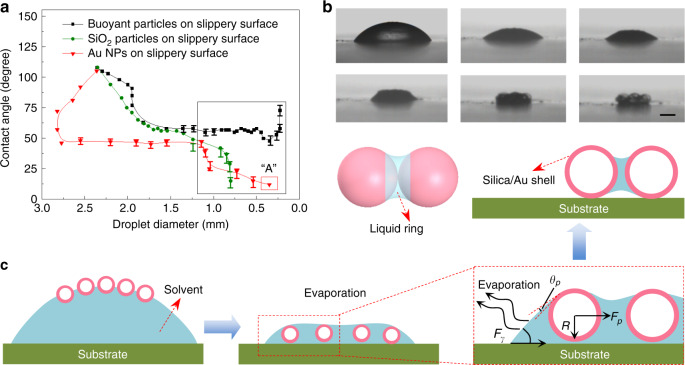
Evaporation processes and mechanism. **a** The relationship between contact angle and droplet diameter for various particulates. The value of droplet diameter marked “A” is estimated owing to the limit of observation. **b** The final drying stage of the evaporation process for a droplet containing buoyant-particulates on slippery surface. The scale bar is 50 μm. **c** Schematics illustration of the close-packed particle aggregation process on a slippery substrate by evaporating a droplet containing floating particles. The inset figure displays the light-weight particle in the vicinity of the leading edge of the thin film.

To analyze the aggregation effect of the buoyant particulate, and the lifting and enriching effects of the solvent and probe molecules, we evaluated the influences of various suspended particulates on the drying process of a droplet on the slippery surface. We compared three types of particulates, i.e., Au NPs, solid SiO_2_ particles, and hollow SiO_2−_ coated by Au nanoparticles. During the evaporation process, the initial contact angles of droplets with different suspended particulates were not significantly different (Fig. 3a). As the evaporation proceeded, the main differences were the changes in the contact angle and contact line (Fig. 3a). When a droplet contained solid silica particulates, the contact line and contact angle gradually decreased (Fig. 3a and Supplementary Fig. 9), finally forms a coffee-ring pattern. In the situation of Au-NP suspended droplet (Supplementary Fig. 10), after a rapid decrease in the contact angle at the early stage, a constant contact angle (CCA) of approximately 46°, was formed. However, after the diameter of the droplet shrinks to less than 1.0 mm, the contact angle gradually reduced to approximately 12° in the end, and the droplet diameter decreased continuously (Fig. 3a). Finally, the resulting aggregated patterns (Supplementary Fig. 11) exhibited the character of a micrometer-sized coffee-ring.

The aggregation processes of the buoyant-particulate suspended droplets on the slippery surface were observed through video microscopy and contact angle measurements (Supplementary Movie 1 and Supplementary Figs. 12 and 3b). As shown in Fig. 3a, after a rapid decrease, a CCA of approximately 58° appeared. More interestingly, the contact angle even increased at the final stage (Fig. 3a, b), which could be critical for the aggregation effect. From the images of final aggregates shown in Supplementary Fig. 13, we obtained direct evidence that the coffee-ring phenomenon had been completely prevented and that a dense-packed pattern had been finally achieved.

To understand the aggregation mechanism of buoyant-particulate, and the lifting and enriching effects of the solvent and containing molecules, an analytical model based on the force analysis of suspended particulates at three-phase (air–water–substrate) interfaces (Fig. 3c) and the Young–Laplace equation has been developed (see details in Supplementary Information). According to the theoretical results, an enrichment and spatial localization mechanism of target molecules is proposed and schematically illustrated in Fig. 3c. Both the driving force to enforce the aggregation of buoyant-particulates and to lift the final solvent from the slippery substrate can be significantly affected by the particle size. A larger floating-particle size is preferable to obtain a dense-packed pattern, lift the solvent, and localize the analyte into the particle–particle interface. This theoretical prediction has been confirmed by a proof-of-concept experiment as shown in Supplementary Fig. 14, in which an aggregation state cannot be formed when the size of floating-particle less than 20 μm. In fact, previous studies have shown that suspended particles may be aggregated in a more close-packed manner on a hydrophobic surface and with increased *θ*_*R*_^21^.

### Plasmonic sensing properties

**Fig. 4:**
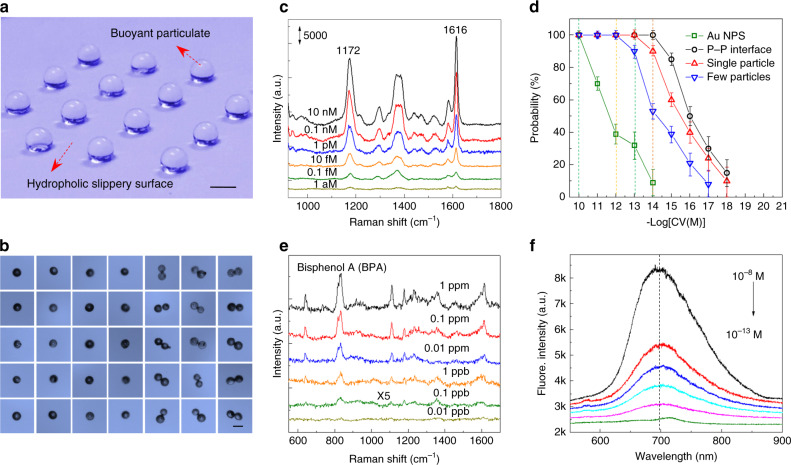
The slippery surface, the plasmonic sensing properties and applications. **a** The droplet with a certain volume on superhydrophobic slippery surface to sort single or dimer particles. The scale bar is 0.5 mm. **b** The optical images of sorting single and dimer buoyant particulate on the top of droplet. The scale bar is 50 μm. **c** SERS spectra of CV molecules obtained from aqueous solutions at different concentrations from 10 nM to 1 aM. **d** Probability of obtaining observable SERS signals at different concentrations for four types of SERS detection protocols, i.e., a droplet containing the Au NPs dries on PTFE porous surface and buoyant-particulate suspended droplet on slippery surface with single, dimer, and few particles. **e** The current SRES strategy for the portable detection of persistent organic pollutants (POPs). **f** Fluorescent spectroscopes and limit of detection of CV probe molecule using the present buoyant particulate protocol.

The current buoyant particulate displays a highly remarkable enrichment capacity of the probe molecules. To exploit the practical applications of this technique, we developed a procedure to trap the buoyant particulates with an accurate sorting of single particles with ∼80% probability (Fig. 4a and Supplementary Fig. 15). Therefore, single, dimer, few particles, and more particle aggregates have been facilely manipulated (Fig. 4b).

The current detection protocol demonstrates an ultrahigh sensitivity for SERS detection. Figure 4c demonstrates SERS spectra using CV molecules as probe molecules, displaying typical Raman peaks of the CV molecules at 1172 and 1616 cm^−1^ bands^22^. The limit of detection (LOD) of the CV molecules using the current SERS protocol can be down to 1 aM (10^−18^ M). Furthermore, using the buoyant particulate-based dimer particle or single-particle-SERS, the 100% probability of collecting observable SERS signal reach the 10 fM or 0.1 pM level. These results represent a remarkable progress compared with other techniques reported in the literature^23^ and are at least 3 or 4 orders of magnitude higher than those using Au NPs as suspended particles (Fig. 4d and Supplementary Fig. 16). According to the calculation of surface area, the enrichment factor is on the scale of ∼10^4^ (Supplementary Fig. 17), owing to the aggregation, enriching, and lifting effects.

As potential applications of the current SERS strategy, we examined the LOD in various dye molecules, e.g., rhodamine 6G, rhodamine B, and malachite green, which are typical illegal additives in realistic food. Using the buoyant particulate-based dimer-particle-SERS protocol, an ultralow LOD, i.e., at 10–100 fM, can be obtained for these molecules (Supplementary Fig. 18). Furthermore, we evaluated the inspection in persistent organic pollutants (POPs), which are typically weakly adsorbed molecules and extensively emerging contaminants in many ground and surface waters. Bisphenol A (BPA), 2,4-dichlorophenol, and naphthalene were selected as target molecules (Supplementary Fig. 18). Figure 4e shows the Raman spectra of the BPA molecule. The LODs for the BPA molecules could reach to an ultralow concentration of ∼0.1 ppb. This sensitivity is much better than those of other SERS protocols from the literature for the molecule detection of POPs^24^. Therefore, owing to the availability of portable Raman spectrometers, the present SERS strategy will be applicable to the rapid in-situ analysis of safety in food manufacturing, environment pollutants, etc., as only a few minutes are required in an optimized process (Supplementary Table 1). Furthermore, we can improve the sensing performance by optimizing the materials and methodology (Supplementary Figs. 19–24). For example, the signal repeatability can be improved by optimizing the sizes of the laser spot and laser power (Supplementary Fig. 23). Furthermore, the sensitivity can also be improved by changing the irradiation laser wavelength and the size of Au NPs (Supplementary Fig. 24).

The advantage of the current buoyant particulate protocol can be also found in other plasmonic nanosensors, e.g., fluorescent sensing. In Fig. 4f, the fluorescent signals can be distinguished even when the CV concentrations are decreased from 10^−11^ to 10^−13^ M. The LOD is determined to be as low as 10^−12^ M. By contrast, the fluorescent images of Au NPs as suspended particulates were difficult to identify even for the CV concentration of 10^−9^ M (Supplementary Fig. 25).

## Discussion

In conclusion, we demonstrated a new buoyant particulate-based few-to-single particle-plasmonic sensing strategy, consisting of light-weight buoyant particulates, a solvent droplet containing analytes, and a slippery surface. During drying, the buoyant particles floated on the top of the solvent, thereby significantly reducing their chances of being pinned on a slippery surface. A large buoyant particulate can be favorable to enrich the solvent and analyte through the aggregation and lifting effects. Therefore, the resulted dense-packed pattern, particularly for dimer or single particles, may significantly increase the sensitivity of plasmonic sensors with an enrichment factor of up to ∼10^4^. Combined with the developed procedure using a superhydrophobic surface to accurately manipulate single particles, the current buoyant particulate strategy is believed to be applicable to a wider range of sensing devices, such as fluorescent, Raman, and infrared spectroscopes for a cost-effective, simple, fast, flexible, and portable detection. Additionally, the strategy is opening new possibilities for a wide range of applications not only in plasmonic enhanced spectroscopes, but also in biological sensors, printing, photonic crystals, complex assemblies, and other devices^25^.

## Methods

### Synthesis

As the schematic shown in Supplementary Fig. 1, the silica-coated Au shell particles were prepared by a seed-mediated growth method, and the detailed procedure could be regarded as the following steps. (i) The chemical modification of hollow SiO_2_ microspheres: 0.1 wt% aqueous hollow SiO_2_ microspheres were prepared by mixing 0.1 g of silica powder with 100 mL of ultrapure water. Next, 50 mL hollow SiO_2_ microspheres, 0.2 mL 0.4% (3-Aminopropyl)trimethoxysilane (APTMS), and 50 mL ultrapure water were mixed under stirring for 24 h, and was further heated at 80 °C. Excess APTMS was removed by centrifuging and re-dispersing in water for 3 times, and the obtained sample was termed as SiO_2_–NH_2_. (ii) The adsorption of Au NPs on hollow SiO_2_ microspheres: Spherical gold NPs with 23 nm in diameter, served as the nanoseeds, were synthesized based on a modified citrate reduction approach^26^, characterized by the scanning electron microscope (SEM) image and UV–vis spectra in Supplementary Fig. 2b. Then, an appropriate amount of SiO_2_–NH_2_ was injected dropwise to the gold seeds solutions. Under vigorous magnetic stirring, almost all of hollow silica microspheres could be covered with Au nanoseeds after 6 h, denoted as SiO_2_–Au nanoseeds. The excess gold seeds were removed by centrifuging in 7000 rpm. SEM images in Supplementary Fig. 2c demonstrated a uniform distribution of Au seeds on SiO_2_ surface. (iii) The synthesis of hollow silica-coated Au shell particles: Firstly, gold hydroxide was obtained by mixing 100 mL H_2_O, 4 mL HAuCl_4_ (10 mM), and 50 mg K_2_CO_3_ powder in dark for 24 h. Then, 2 mL aqueous SiO_2_–Au nanoseeds was added into 5 mL gold hydroxide solutions under vigorous stirring to prevent the up-floating of microsphere. After 5 min, 0.15 mL NH_2_OH·HCl (0.1 M) solution was added dropwise, and the Au shell coating would be finished 3 h later. In order to obtain more uniform coverage of Au shell, a spot of AgNO_3_ solution was brought into the reaction system. As shown in Supplementary Fig. 3, homogeneous and closely packed gold shell was prepared. The gold NPs were ∼57 nm with the particle–particle gaps for only several nanometers.

### Preparation of SERS substrates

We used the hollow silica-coated Au shell particles as SERS-active materials, and a hydrophobic slippery Teflon membrane as support surface. The fabrication of slippery membrane was as following: the Teflon membrane was fixed onto a flat glass slide (5 cm × 5 cm) by double-sided adhesive. Then, 0.5 mL perfluorinated lubricant (Dupont, GPL 105) was dispersed on the membrane by spin coating, and thus was heated at 90 °C for 30 min. For analytes detection, such as crystal violet, 50 μL aqueous solution of probe molecules and 10 μL silica-coated Au shell particles were dropped onto the hydrophobic slippery surface at 150 °C. During the drying process, we observed that the particles floated on the surface of droplets like a “boat” due to the ultra-low density of hollow SiO_2_, as shown in Fig. 1. The closed packaged few-particles aggregate would be obtained after solvent evaporation (shown in Supplementary Fig. 13), and then was used as SERS substrates.

### Raman and fluorescence spectroscopy

The Raman spectra, fluorescence spectra, and fluorescence imaging measurements were performed in a confocal microscope-based Raman spectrometer on a home-made optical testing platform in our lab. For SERS test, the samples were excited by a 633 nm laser with ∼0.2 mW, and the acquisition time was 30 s. For fluorescence test, the signal was collected by a 532 nm laser with the laser power of ∼5 mW. The acquisition time was 60 s. In addition, the fluorescence imaging was obtained by the excitation of a mercury lamp.

### The characterizations of buoyant particulates

The morphology and structure of the products were characterized using a scanning electron microscope (JEOL, JSM-7000F). The optical properties were characterized using ultraviolet–visible spectroscopy (Agilent, Carry 60). The contact angle was measured on an optical contact angle instrument (KRUSS, DSA 100).

## Supplementary information

Supplementary Information

Peer Review File

Description of Additional Supplementary Files

Supplementary Movie 1

Supplementary Movie 2

Supplementary Movie 3

Supplementary Movie 4

## Data Availability

Research data supporting this publication is available at 10.1038/s41467-020-16329-y. The source data underlying Figs. 2e, 3a, and 4c–f and Supplementary Figs. 2b, 3c, 18a–f, 19, 20b, 23a, b, 24b, c, and 25c are provided as a Source Data file. Other data are available from the corresponding author upon reasonable request.

## Source data

Source Data
