# Supplementary material for: Buoyant particulate strategy for few-to-single particle-based plasmonic enhanced nanosensors
Source: Nat Commun. 2020 May 25;11:2603. doi: 10.1038/s41467-020-16329-y (PMC7248072; doi:10.1038/s41467-020-16329-y)
Supplement: Supplementary file 1 — Supplementary Information [file 41467_2020_16329_MOESM1_ESM.pdf]

Supplemental Information for "Buoyant particulate strategy for few-to-single particle-based plasmonic enhanced nanosensors" byZhang *et al.*

Buoyant particulate strategy for few-to-single particle-based plasmonic enhanced nanosensors

Dongjie Zhang[1,#], Leqin Peng[2,#], Xinglong Shang[2], Wenxiu Zheng[2], Hongjun You[1], Teng Xu[3], Bo Ma[3], Bin Ren[4,*], Jixiang Fang[1,*]

[1]Key Laboratory of Physical Electronics and Devices of Ministry of Education, School of Electronic and Information Engineering, Xi'an Jiaotong University, Xi'an, Shann xi, 710049, P. R. China

[2]State Key Lab of Multiphase Flow in Power Engineering, Xi'an Jiaotong University, Xi'an, Shann xi, 710049, P. R. China

[3]Single-Cell Center, CAS Key Laboratory of Biofuels and Shandong Key Laboratory of Energy Genetics, Qingdao Institute of BioEnergy and Bioprocess Technology, Chinese Academy of Sciences, Qingdao, Shandong 266101, P. R. China

[4]State Key Laboratory of Physical Chemistry of Solid Surfaces, Department of Chemistry, College of Chemistry and Chemical Engineering, Xiamen University,361005 Xiamen, P. R. China

[*]E-mail: jxfang@mail.xjtu.edu.cn, bren@xmu.edu.cn

# These authors contributed equally to this work.

Contents

Supplementary Figures

Supplementary Tables

Supplementary Notes

Supplementary References

Supplementary Figures

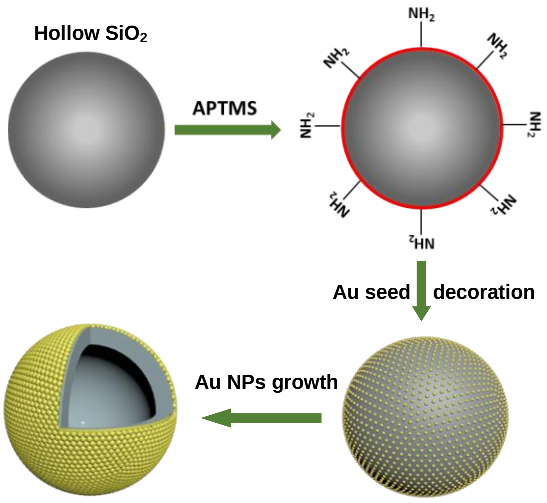

Supplementary Fig. 1 | Schematic procedure for the preparation of buoyant SiO_2-Au core-shell particulate.

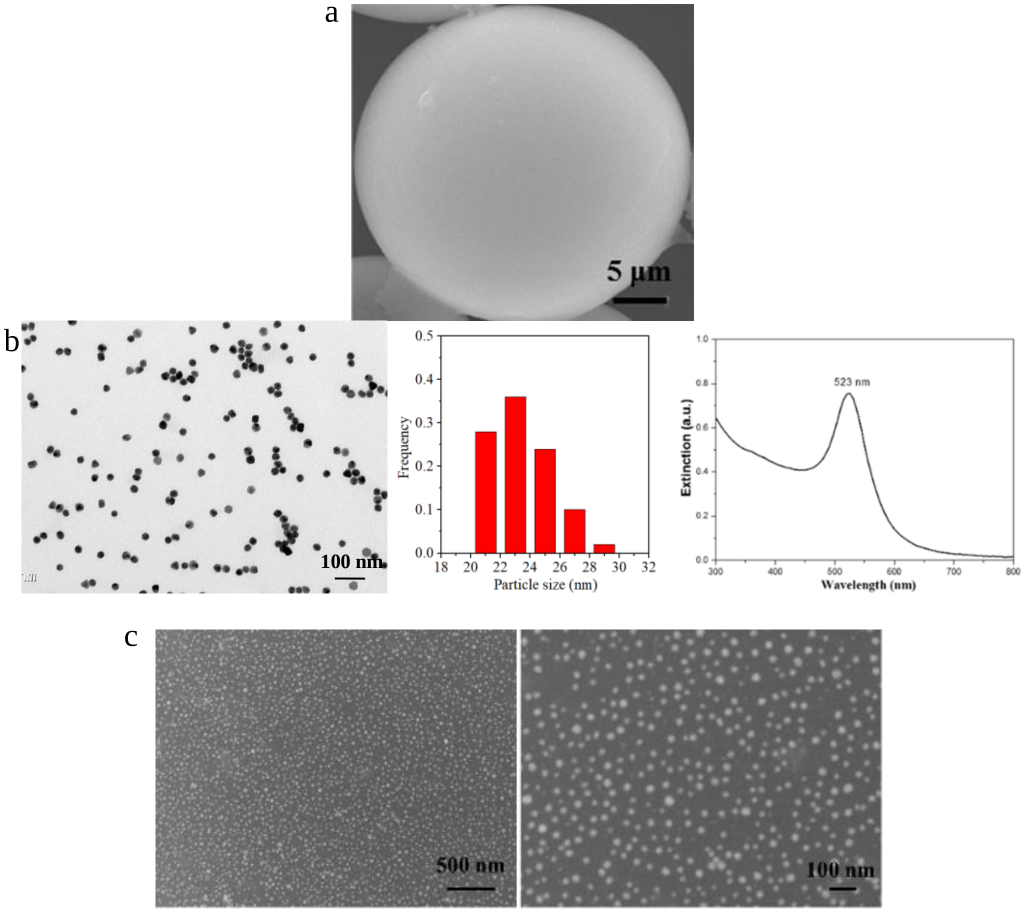

Supplementary Fig. 2 | (a) The SEM image of hollow SiO_2 particles. (b) The TEM image, the size distribution and the UV-vis spectrum of Au seeds. (c) The SEM images of colloidal Au seeds decorated on hollow SiO_2 surface.

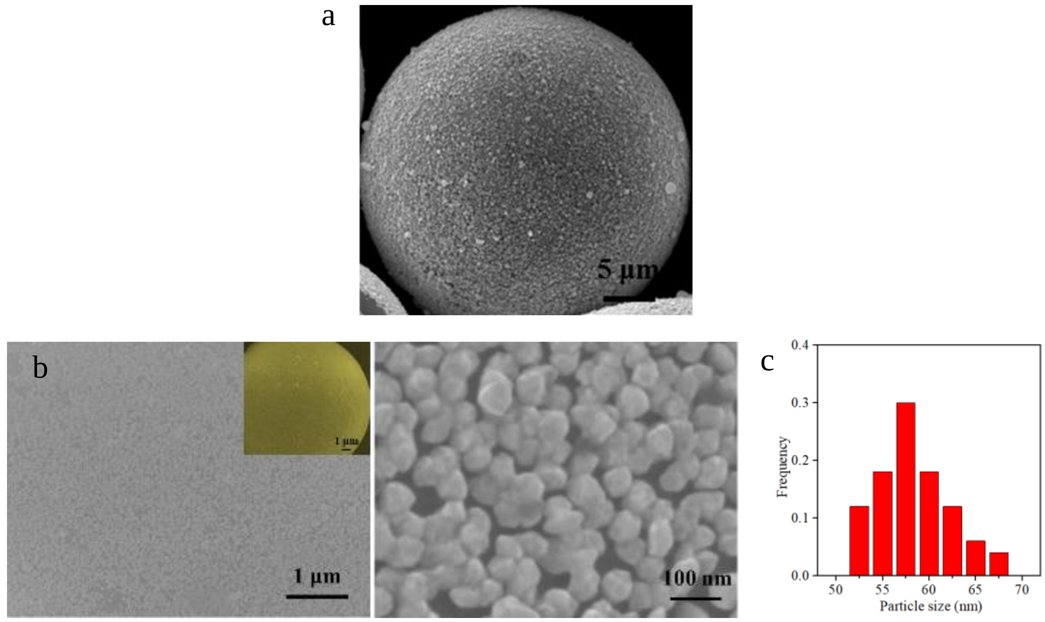

Supplementary Fig. 3 | (a)-(b) The SEM characterizations of the buoyant SiO_2-Au NPs particulate, where Au NPs grew on SiO_2 surface. (c) The size distribution of Au nanoparticles, which is around 57 nm.

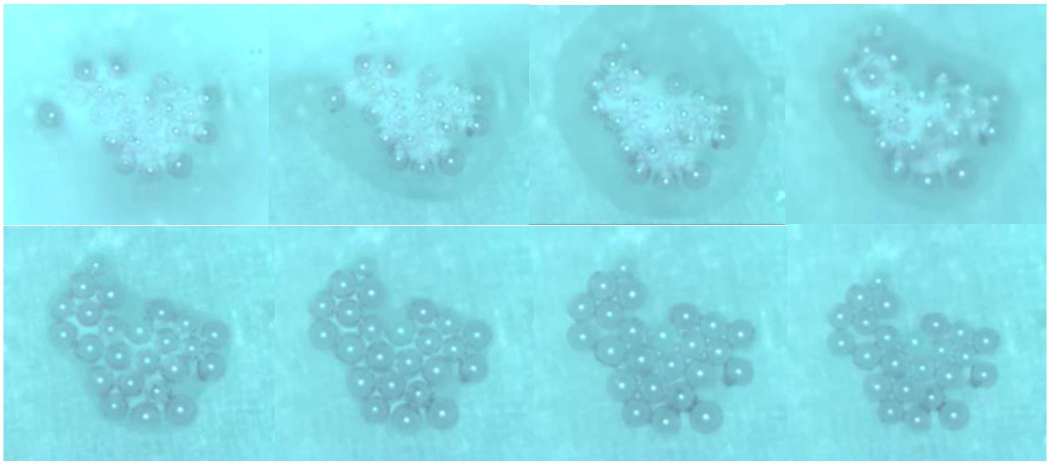

Supplementary Fig. 4 | The aggregation process of floating particles distilled from Supplementary Video 2.

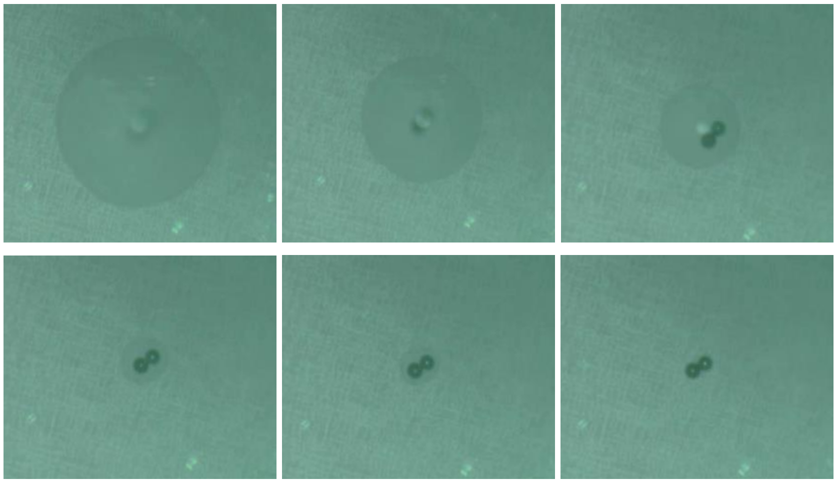

Supplementary Fig. 5 | The aggregation process of floating particles distilled from Supplementary Video 3.

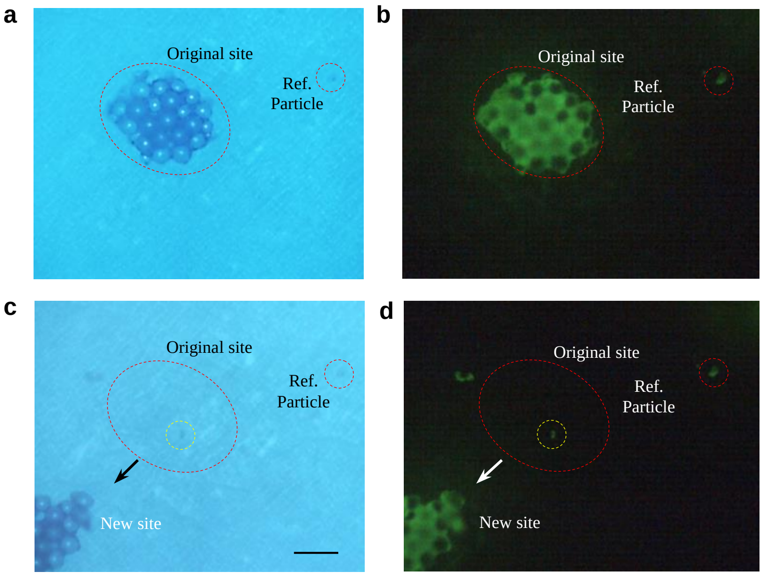

Supplementary Fig. 6 | Optical and fluorescent images of aggregate using hollow SiO_2 coated with Au nanoparticles before (a) – (b) and after (c) – (d) moved. The concentration of CV is 10^{-8} M. The red circles show that after we moved the aggregate, the original site does not display the 'network'-shaped fluorescent image. The scale bar is 100 μm.

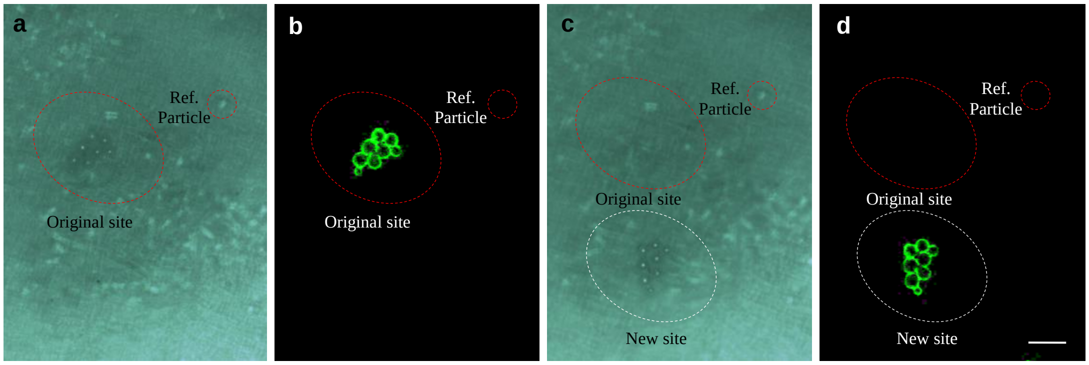

Supplementary Fig. 7 | Optical and fluorescent images of aggregate using hollow SiO_2 particles before (a) – (b) and after (c) – (d) moved. The concentration of CV is 10^{-8} M. The red circles show that after we moved the aggregate, the original site does not display the ‘network’-shaped fluorescent image. The scale bar is 100 μm.

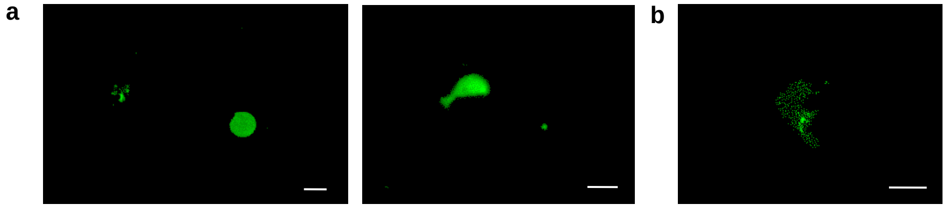

Supplementary Fig. 8 | Fluorescent images of aggregate after a droplet evaporation on the slippery surface containing the CV molecules with concentration of (a) 10^{-8} M and (b) 10^{-9} M. The detectable fluorescent signal can still be observed at a CV concentration of 10^{-9} M. The scale bar in **a** is 100 μm and in **b** is 50 μm.

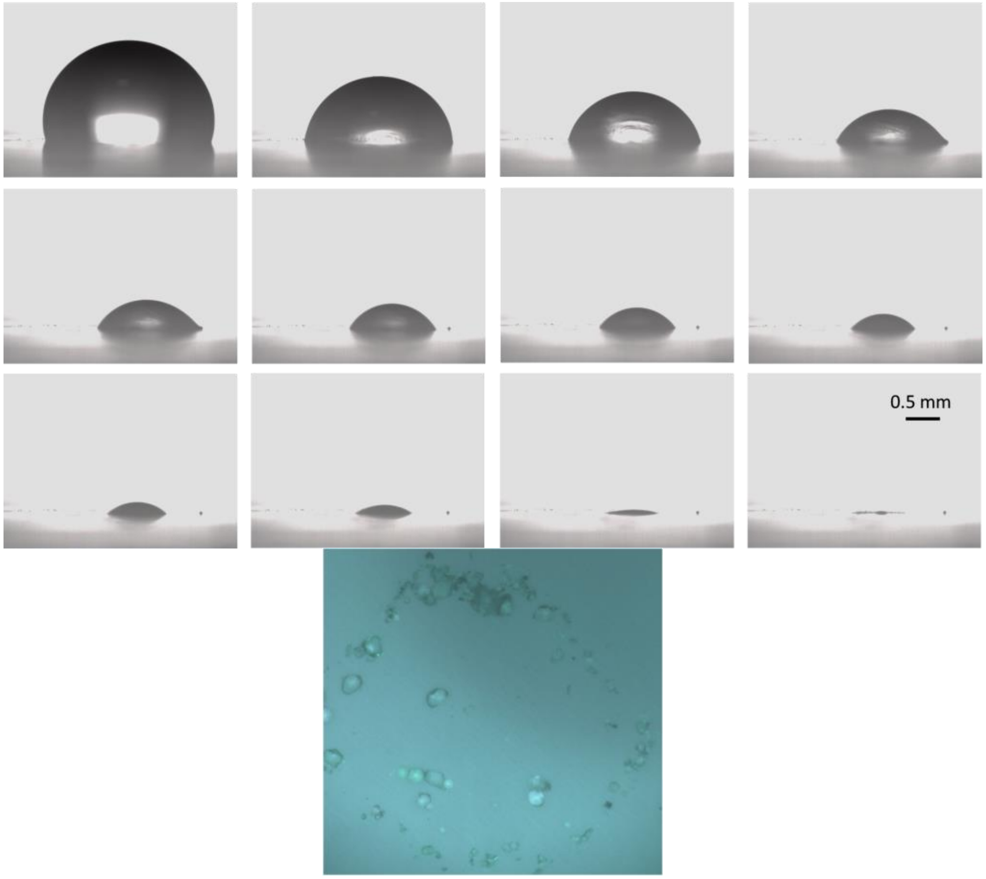

Supplementary Fig. 9 | Drying process of solid SiO_2 particles on a hydrophobic slippery surface. The bottom optical image shows the dispersed particle distribution.

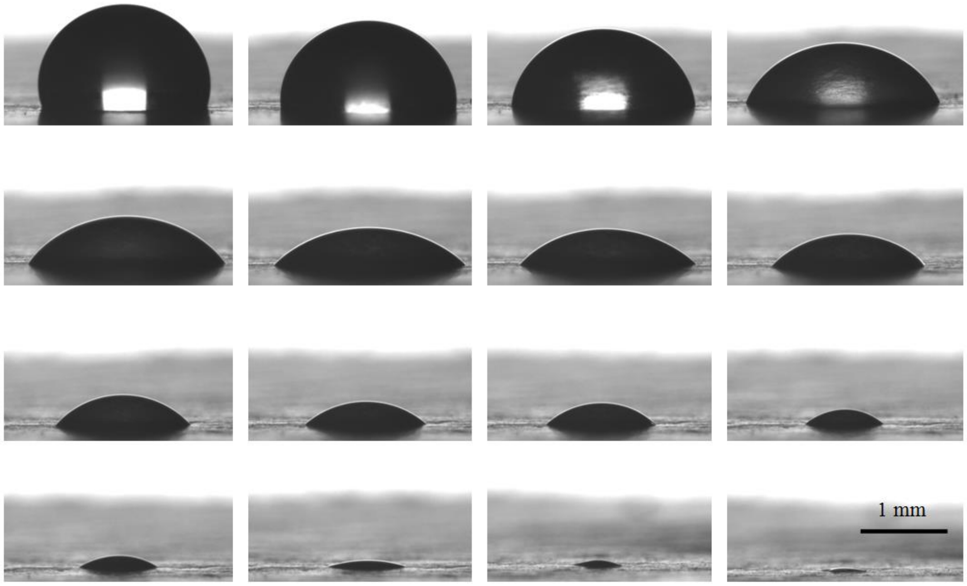

Supplementary Fig. 10 | Drying process of Au colloids on hydrophobic slippery surface. The images show the time evolution of contact line and contact angle.

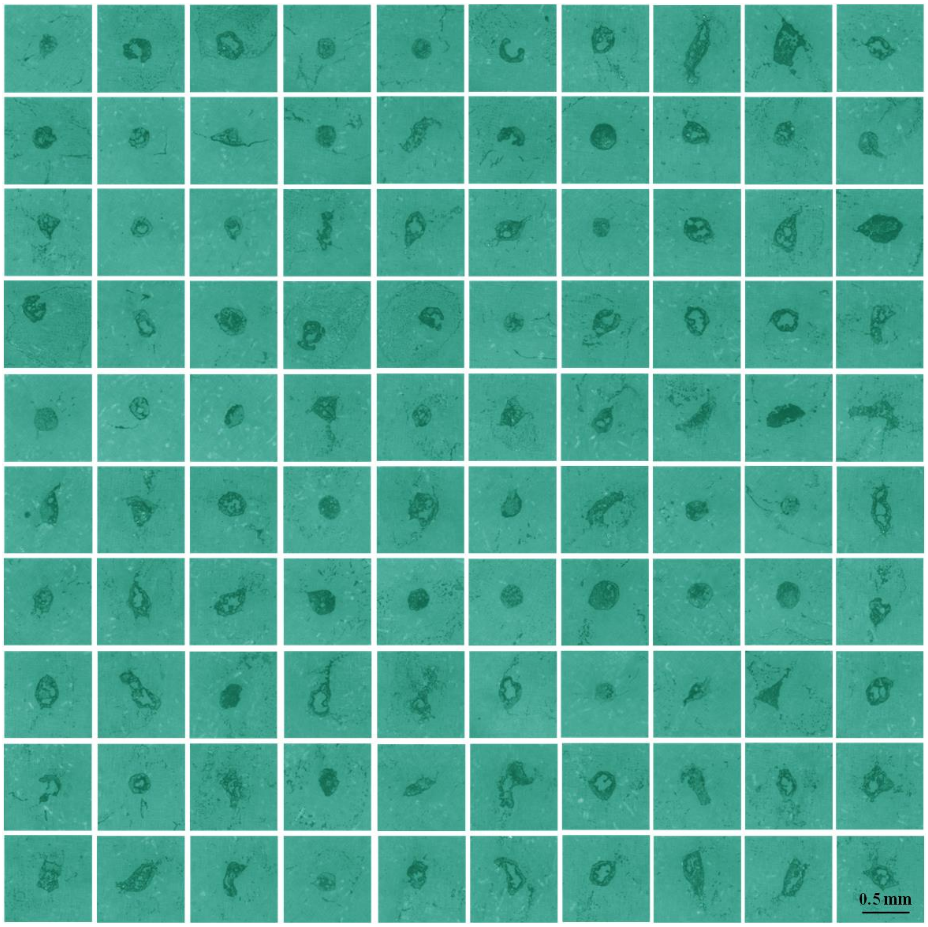

Supplementary Fig. 11 | Collective 100 images of Au colloidal aggregation drying on a hydrophobic slippery surface.

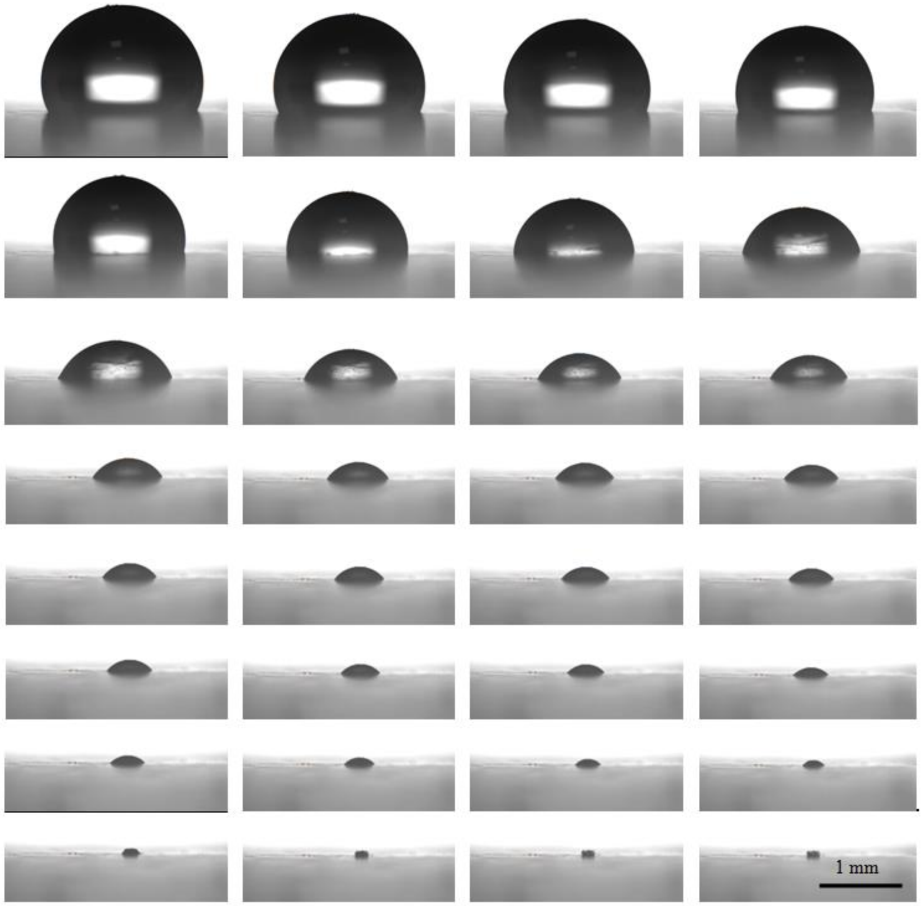

Supplementary Fig. 12 | Drying process of buoyant particulate on a hydrophobic slippery surface. The images show the time evolution of contact line and contact angle.

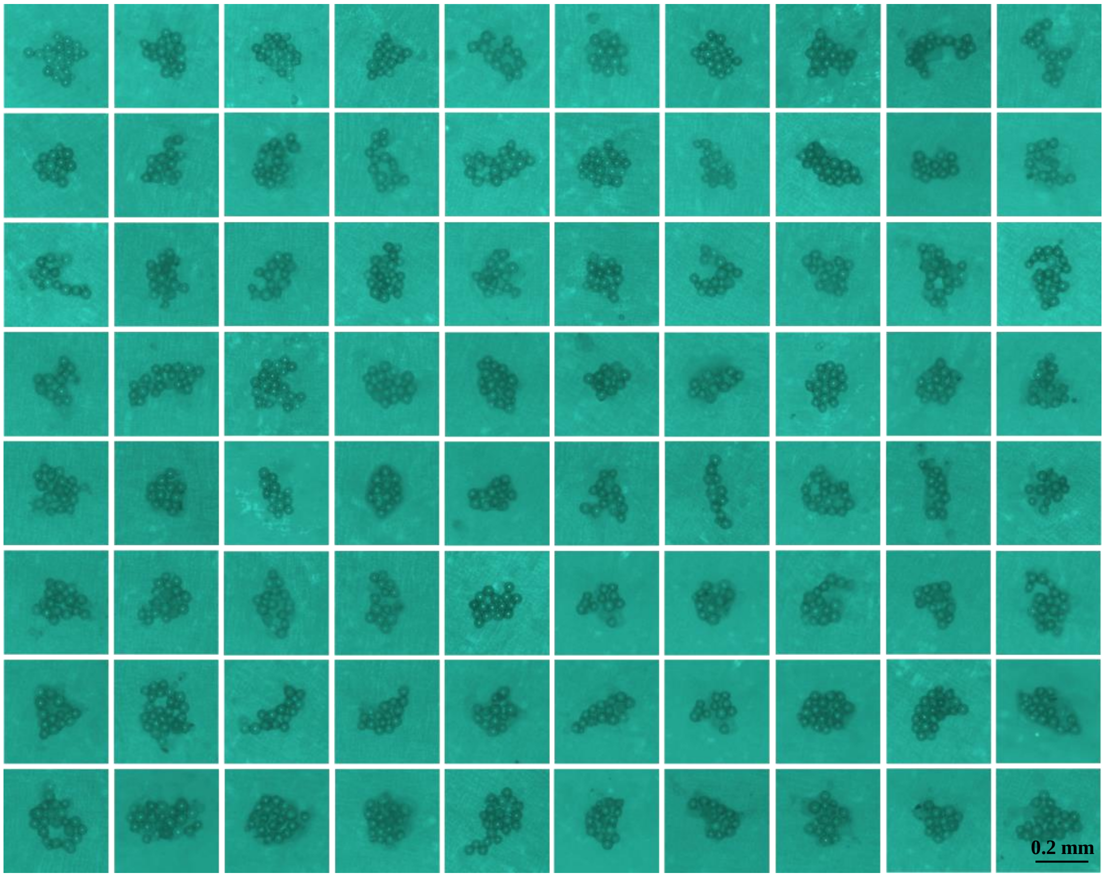

Supplementary Fig. 13 | Collective 80 images for 10-20 buoyant particulate after drying on a hydrophobic slippery surface.

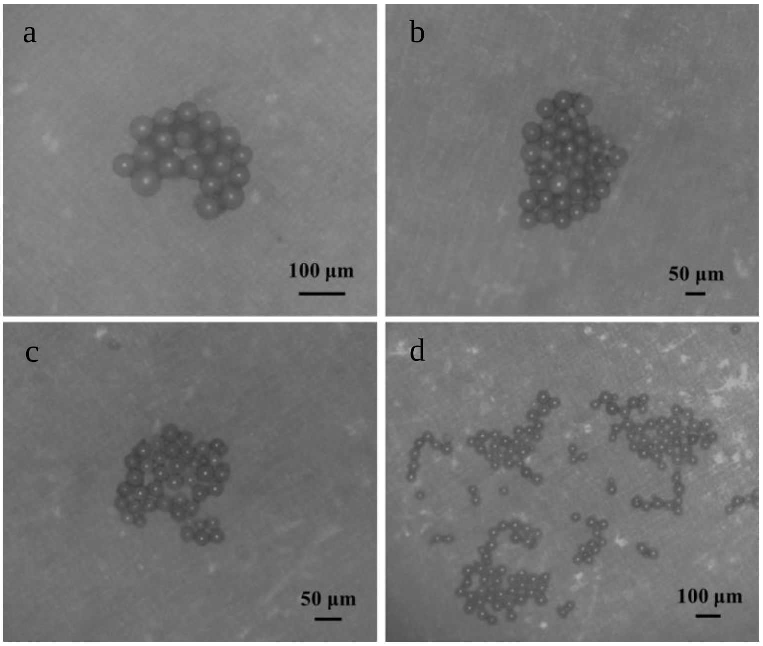

Supplementary Fig. 14 | Optical images of aggregated buoyant particulates with various sizes: (a) 55 µm; (b) 41 µm; (c) 30 µm and (d) 20 µm.

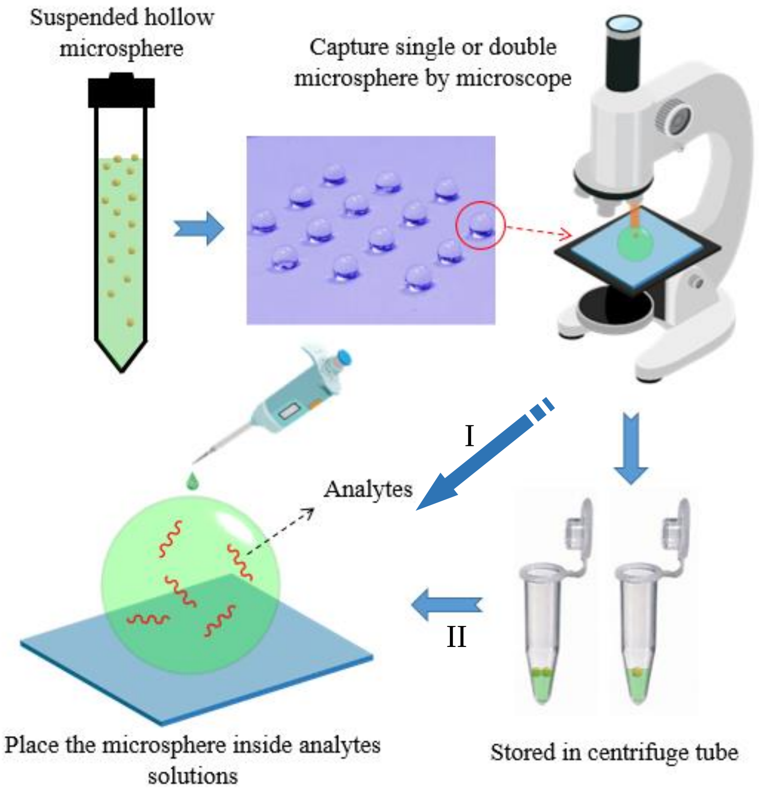

Supplementary Fig. 15 | Manipulation procedure of sorting single buoyant particle by superhydrophobic slippery substrate. **Route I**: after sorting of the particle by hydrophobic surface, then confirming the amount by microscope, and directly adding the solvent containing analyte to further detect. **Route II**: after sorting and confirming the amount of the floating particles. The particles and few solvent are stored in a container for the usage when detection.

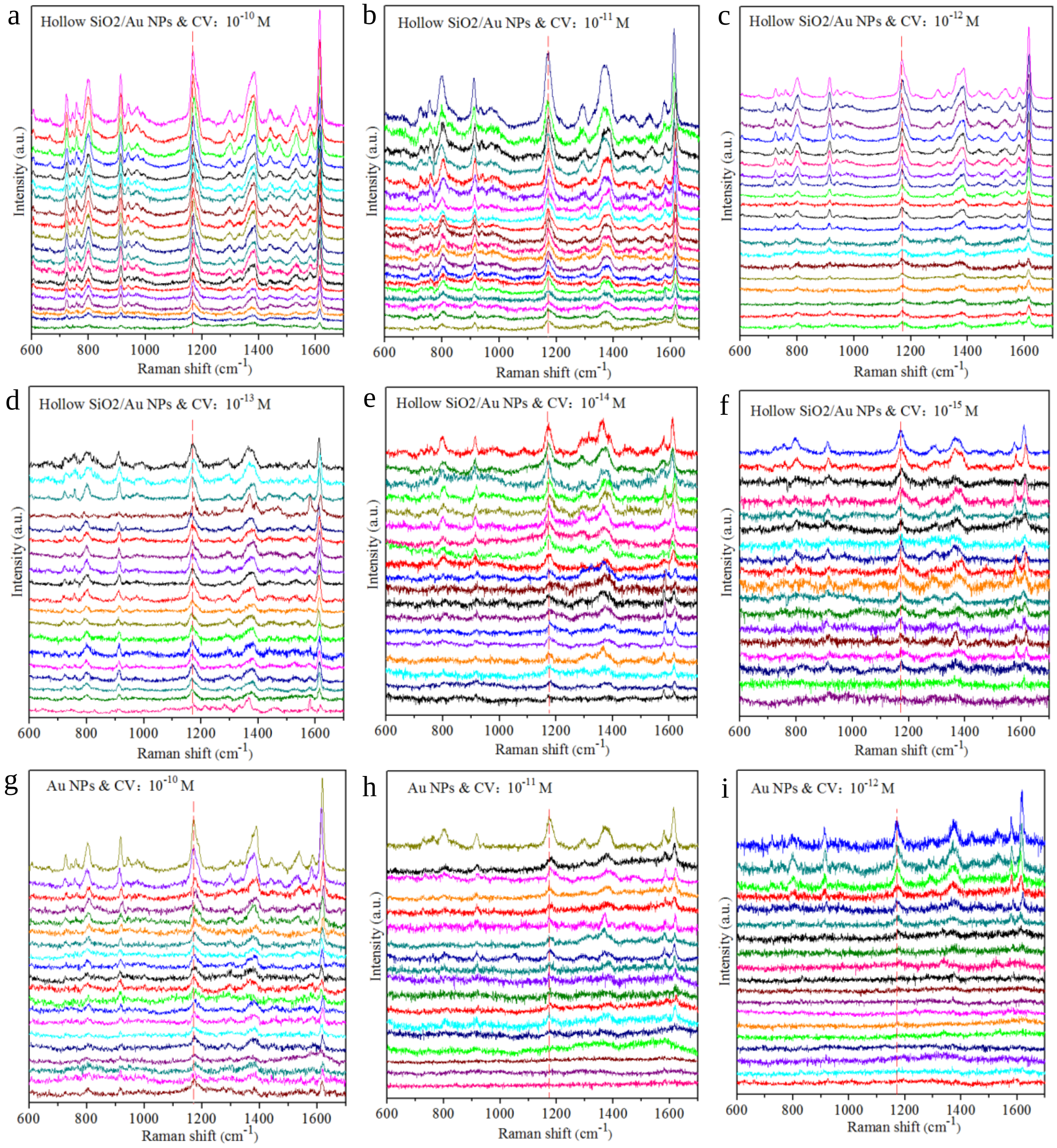

Supplementary Fig. 16 | Compared SERS measurements of diluted CV molecule with different concentrations: (a-f) Hollow SiO_2/Au microsphere on a hydrophobic slippery surface; (g-i) Au NPs on a hydrophobic slippery surface.

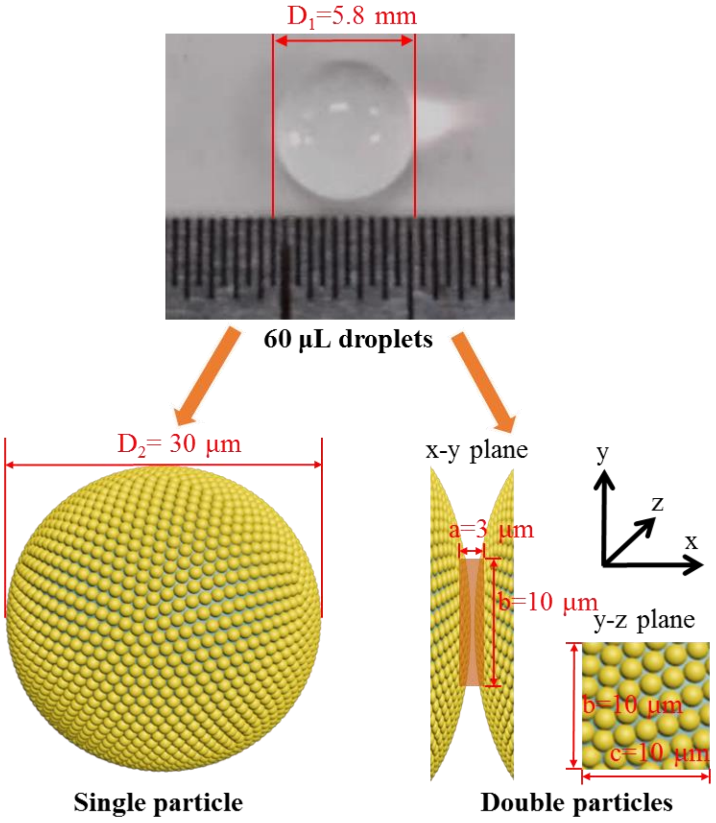

Supplementary Fig. 17 | Geometric model for the evaluation of enrichment factor.

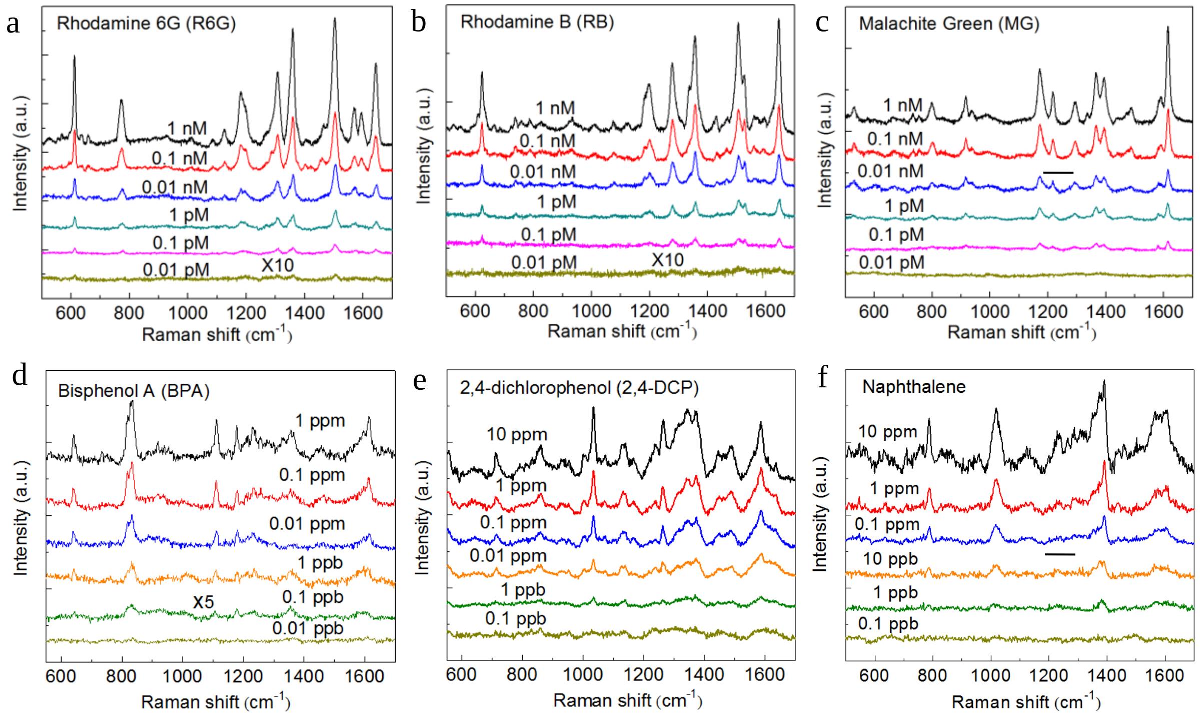

Supplementary Fig. 18 | The SERS detection using current SRES strategy for diverse molecules: (a) Rhodamine 6G, (b) Rhodamine B, (c) Malachite green, and POPs molecules including (d) bisphenol A, (e) 2, 4-dichlorophenol, and (f) naphthalene.

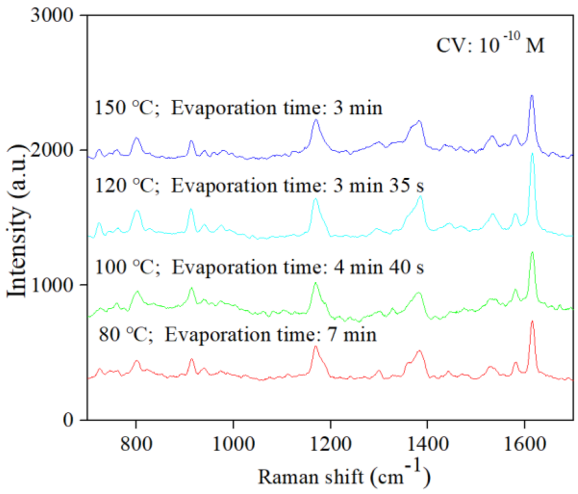

Supplementary Fig. 19 | The SERS properties as a function of heating temperature during the evaporation of a droplet containing the buoyant SiO_2-Au NPs particulate. Above curves indicate that as the increase of heating temperature, the SERS performance don't change remarkably. However, by means of increasing the heating temperature, the operation and detection period can be significantly decreased for the real-time rapid analysis.

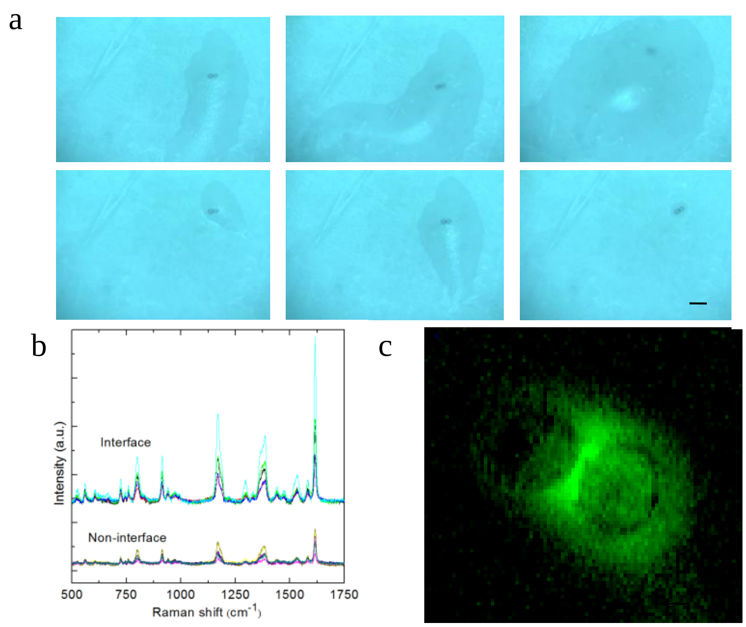

Supplementary Fig. 20 | Last evaporation period of dimer floating particles on a hydrophobic slippery surface with some defects such as scratch or contamination. (a) Optical images; (b) SERS spectra collected on the interface and non-interface regions; (c) fluorescent images. It is noted that the Teflon membrane uniformity, lubricant and the solvent types could be the significant factors to obtain a highly localized molecule enriching effect. The scale bar is 100 μm.

a

b

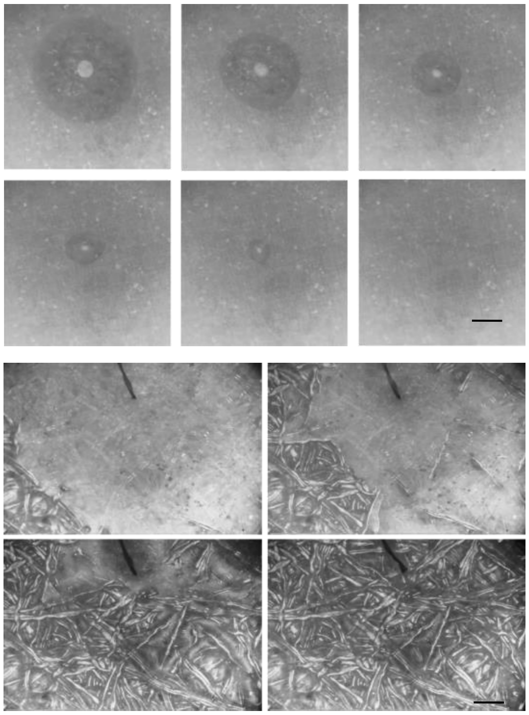

Supplementary Fig. 21 | The influence of Teflon membrane on the aggregation of solvent. The pore sizes were: (a) 0.1 μm and (b) 0.22 μm. We found that with a small pore size e.g. 0.1 μm, the enrichment effect shows excellent, and with a large pore size e.g. 0.22 μm, the droplet aggregation is not very efficient, and showing a large area distribution of the molecule. The scale bars in **a** is 100 μm, and in **b** is 50 μm.

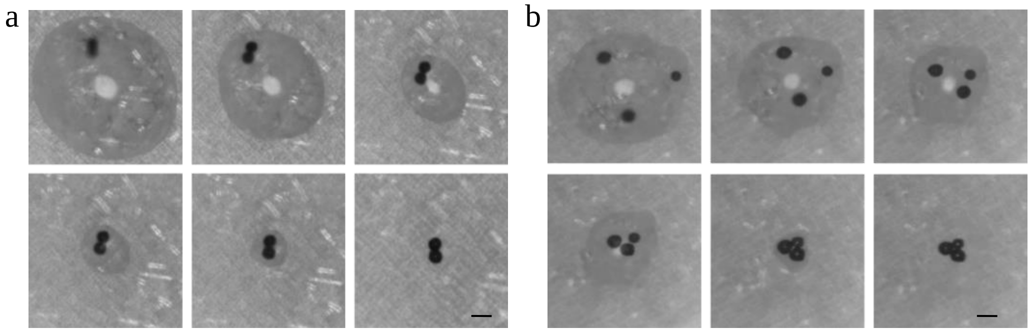

Supplementary Fig. 22 | The influence of lubricant type and amount on enrichment effect. In the experiments, we have optimized the lubricant by using various types of Perfluorinated lubricant: **a**, (Dupont, GPL 105) and **b**, (Dupont, GPL 103). From the results, no significant influence has been found, and with GPL 103, the slippery capacity can be increased. For the amount of the lubricant, if less than a critical value, the slippery ability decreases, the enrichment effect would be worse. The scale bars in **a** and **b** are 50 μm.

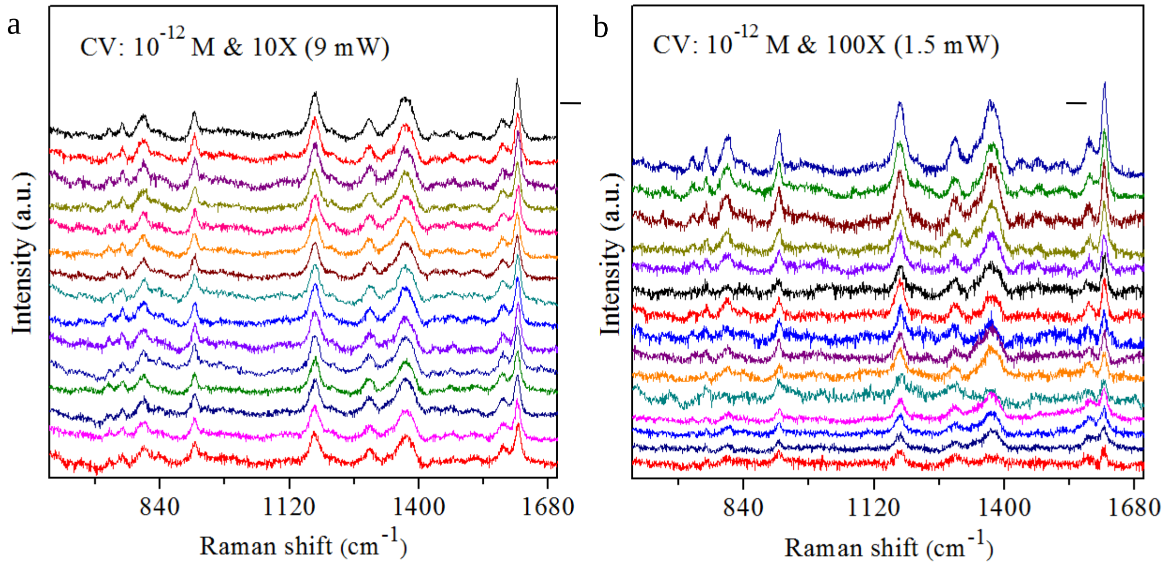

Supplementary Fig. 23 | The Raman spectra with current buoyant particulate-based SERS protocol under various laser spot sizes and powers: (a) 10x lens and (b) 100x lens.

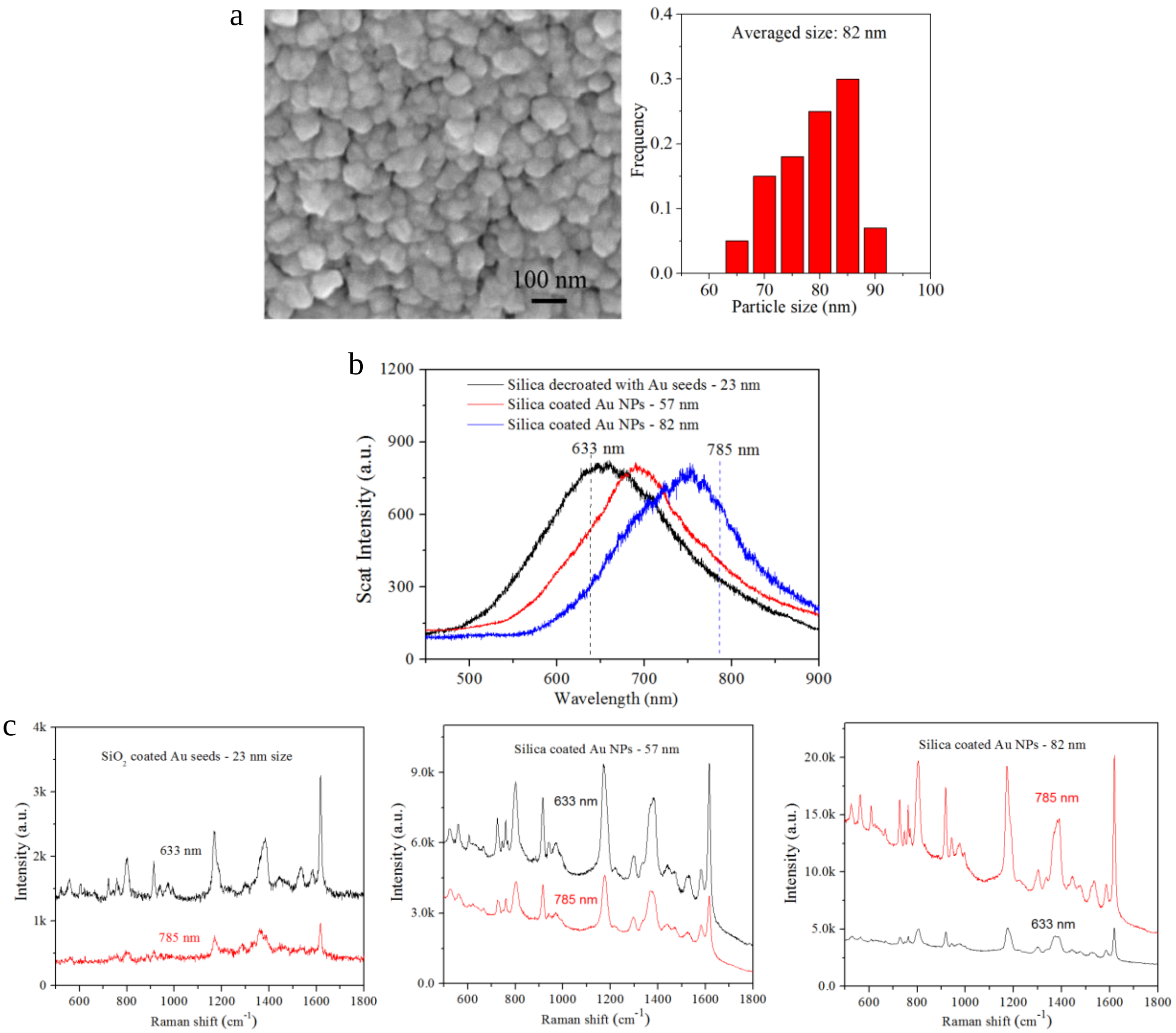

Supplementary Fig. 24 | (a) SEM characterizations of the buoyant SiO_2-Au NPs particulate, where Au NPs grew on hollow SiO_2 surface. The size of Au nanoparticles is around 80 nm. (b) The scattering spectra for silica decorated with Au seeds, and further grew Au NPs with sizes of 57 nm and 82 nm. (c) The Raman spectra for above three particulates under 633 nm and 785 nm laser irradiation.

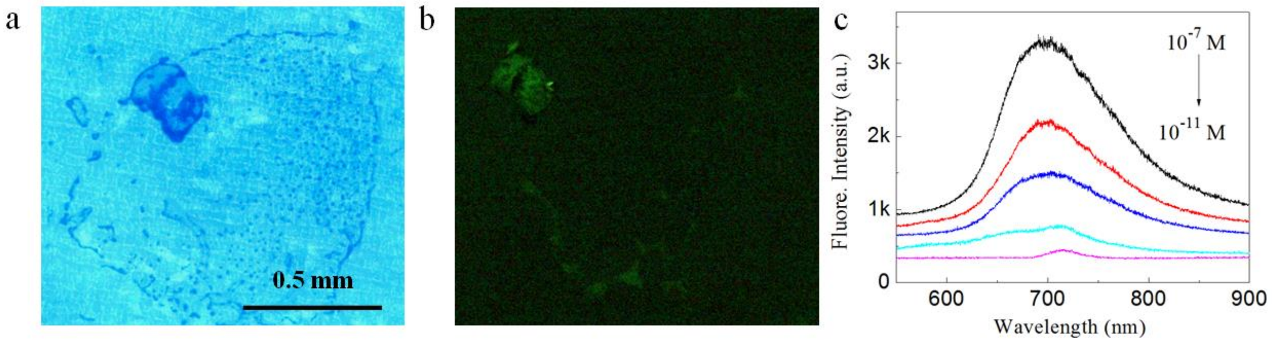

Supplementary Fig. 25 | The enriching effect of Au NPs on slippery surface. (a) optical and (b) fluorescent images of Au NPs at CV concentration of 10^{-9} M; (c) fluorescent signals of CV with concentrations from10^{-7} M to 10^{-11} M.

Supplementary Tables

Supplementary Table 1. Evaporation speed of aqueous and ethanol solvent with 60 μL in volume.

Heating Temperature	Aqueous solution of CV molecules	Ethanol solution of CV molecules
150℃	15 min	3 min
120℃	19 min	3 min 35 s
100℃	27 min	4 min 40 s
80℃	47 min	7 min

Supplementary Notes

Supplementary Note 1: Calculation of solvent and molecules enrichment factor.

The enrichment efficiency of solvent and molecules on the hydrophobic slippery surface was evaluated based on the changes of solvent surface area during evaporation. Firstly, we estimated the surface area of the initial droplet with 60 μL in volume. As shown in Supplementary Fig. 18, the drop was ~5.8 mm in diameter, and thus the surface area S_1 could be obtained as following:

$$S_1 = \frac{1}{4}\pi D_1^2 = \frac{1}{4} \times 3.14 \times (5.8)^2 \ \ mm^2 = 2.5 \times 10^7 \ \ \mu m^2$$

After the droplets drying on the hydrophobic slippery substrate, almost all solvent and molecules were concentrated on the surface of single particle or into the interface region of dimer particles. In case of single buoyant particle (Supplementary Fig. 18), we assumed that all of solvent and probe molecules were concentrated on the surface region of single particle according to the experiment results. Thus, the surface area S_2 could be obtained as following:

$$S_2 = \pi D_2^2 = 3.14 \times (30)^2 \ \ \mu m^2 = 2826 \ \ \mu m^2$$

In case of dimer buoyant particle, most solvent and analytes were concentrated into the gaps region according to the fluorescence imaging in Fig.2a. We assumed that all molecules were guided into the marked region shown in Fig. 2c and Supplementary Fig. 7. The surface area S_3 could be calculated as following:

$$S_3 = 2 \times (a \times b + b \times c + a \times c) = 2 \times (3 \times 10 + 10 \times 10 + 3 \times 10) \ \ \mu m^2 = 320 \ \ \mu m^2$$

Lastly, the enrichment factors of solvent and molecules for single particle and double particles were defined as ε_1 and ε_1, which were calculated by the specific value of surface area before and after evaporation. Therefore, we could obtain the final ε_1 and ε_2 as following:

$$\varepsilon_1 = S_1/S_2 = 8.89 \times 10^3$$

$$\varepsilon_2 = S_1/S_3 = 7.14 \times 10^4$$

Supplementary Note 2: Theoretical section

1. To understand the aggregation mechanism of buoyant particulates, we developed an analytical model to describe the influence of floating-particle configurations on droplet evaporation. The model is rooted in the force analysis of the suspended particulate at three phase (air–water-substrate) interfaces (Fig. 3c). For the floating-particle at the final evaporation stage as shown in Fig. 3c, a thin wetting film may be formed between the particle and substrate. The liquid bridge covering particles is formed and the height of liquid surface is close to the particle diameter. Thus the driving force acting on the particle can be written as[1]

$$F_p = 2\pi\sigma R\ (\cos\theta_R + \cos\theta_p) \qquad (1)$$

where θ_p is the contact angle between the liquid surface and particle surface, as shown in Fig. 3c, which is a natural parameter about the substrate, solvent, and atmosphere. For the current system, the values of θ_p and θ_R can be regarded as invariant. Therefore, the driving force acting on the particle is only affected by the particle radius.

In a CCA evaporation mode, it is reasonable to assume that the sliding friction force of the particle is approximately the friction force at the contact line, which is proportional to the value of ($\cos\theta_R$ – $\cos\theta_e$) from the unbalanced Young's stress.[2-3] θ_e is the static contact angle, which is affected by the nature of the substrates and solvent. Thus, when the driving force F_p is equal to the friction force, the critical particle radius R_c for particulate aggregating inwards can be derived as,

$$R_c \propto \frac{(\cos\theta_e - \cos\theta_R)}{(\cos\theta_R + \cos\theta_p)} \qquad (2)$$

From Eq.1 and 2, we note that the critical particle size is determined by θ_e, θ_p and θ_R. For the given system, θ_e, θ_p and θ_R are constant parameters. Hence, a critical particle size, R_c exists. When the size of floating-particle exceeds this value, the liquid capillary attraction F_p may dominate during the aggregating process.

2. The effect of gravity on the capillary bridge, formed in the gap between two spherical particles (Supplementary Fig. 26) in the micron scale, is neglected because the Bond number is approximately 0 (10^{-5}), i.e., significantly less than 1. The bond number (Bo = $\rho g R^2/\sigma$) is a dimensionless number measuring the importance of gravitational forces compared with surface tension forces. The surface tension, σ, between the gas and liquid is constant, indicating a constant mean curvature along the gas-liquid surface. The pendular ring is assumed to be axisymmetric about the connecting center line of two equally sized spheres. The shape of a pendular ring can be described by the Young-Laplace equation in two-dimensional cylinder coordinate (Supplementary Fig. 26) by $r(x)$,

$$\frac{1}{r(1+r'^2)^{1/2}} - \frac{r''}{(1+r'^2)^{3/2}} = -2k \tag{3}$$

where $r' = \partial r/\partial x$, $r'' = \partial^2 r/\partial x^2$. $k = \Delta p/(2\sigma)$, is the characteristic parameter for the shape of the pendular ring, where Δp is the pressure difference across the gas-liquid interface.

The pendular ring is constrained on the particle surfaces with two boundary conditions:

$$r = r_p, \qquad x = 0; \tag{4}$$

$$r' = \cot\left(\theta_p + \beta\right) \quad x = x_c \tag{5}$$

where θ_p is the contact angle, β the filled-angle between the x-axis and the line contacting the contact point and center of the circle, x_c the x-coordinate of the contact point, r_p the r-coordinate with r' =0. Eqn. (3) is transformed to

$$\frac{1}{rr'}\frac{\mathrm{d}}{\mathrm{d}x}\left[\frac{r}{(1+r'^2)^{1/2}}\right]=-2k \tag{6}$$

Integrating with the boundary condition of Eq. (5), Eq. (6) can be rewritten as

$$\frac{r}{(1+r'^2)^{1/2}}=-kr^2+c_0 \tag{7}$$

where $c_0=r_c\sin\left(\theta_p+\beta\right)+kr_c^2$, r_c is the r-coordinate of the contact point. The following result is obtained using the geometrical relationship

$$\frac{1}{(1+r'^2)^{1/2}}=-kr+\frac{R\sin\beta\sin\left(\theta_p+\beta\right)+kR^2\sin^2\beta}{r}=f\left(r\right) \tag{8}$$

For a given k and β, further integration results in the following:

$$x=\int_{r_c}^{r}f\left(r\right)\left[1-f^2\left(r\right)\right]^{-1/2}\mathrm{d}r+x_c \tag{9}$$

Solutions of the profile equation can be obtained as a combination of standard elliptic integrals. Combining Eq. (4) and (7), r_p can be represented as

$$r_p=\left(\sqrt{1+4kc_0}-1\right)\big/\left(2k\right) \tag{10}$$

The volume of the residual pendular ring is given by

$$V=2\pi\int_{r_p}^{r_c}r^2f\left(r\right)\left[1-f^2\left(r\right)\right]^{-1/2}\mathrm{d}r-2\pi\left(R-x_c/3\right)x_c^2 \tag{11}$$

Once the shape of the pendular ring is determined for a certain particle radius R, the filled-angle β, surface tension σ, relative volume of liquid with respect to the solid sphere volume, and the capillary force arising from the interaction between the solid and liquid surface can be obtained. The capillary force comprises two parts: the surface tension term acting at the wetting perimeter, tangent to the meniscus at the intersection with the solid surface; the pressure difference term across the curved gas-liquid interface, which is computed over the axially projected wetting area of each particle.

To identify the capillary rise of the liquid, which locates the void that formed among the landed-particles and substrate, the vertical component of the capillary force is quantified. The vertical component of the capillary force F_r can be expressed as

$$F_r \approx -2\pi r_c \sigma \cos\left(\theta_p + \beta\right) + \Delta p \cdot 2 x_c r_p \qquad (12)$$

where the first item corresponds to the wetting force arising from the contact line and the second one to the Laplace force. The projected area in the vertical direction is assumed as an approximately rectangular projected area of the pendular ring with width $2x_c$ and length r_p. Furthermore, the vertical capillary force can be represented with independent variables as

$$F_r \approx -2\pi\sigma R \sin\beta \cos\left(\theta_p + \beta\right) + 2\sigma R\left(1 - \cos\beta\right)\left(\sqrt{1 + 4kc_0} - 1\right) \qquad (13)$$

Obviously, with a given filled-angle β, the wetting force (first item) is proportional to the particle radius. The unknown parameter in the second item is kc_0. From the above equations, $kc_0 = kR\sin\beta\sin(\alpha+\beta)+(kR\sin\beta)^2$, and the decisive parameter is kR. However, kR is constant at a given filled-angle. Under a given filled-angle β, the profile of the liquid ring is coaxial parallel at various radii. Considering the pressure difference, $kR = (1/r_1+1/r_2)R/2$, where r_1 and r_2 are two principle curvature radii that are both proportional to the particle radius with the coaxial parallel profile. Then, the Laplace force (second item) is also proportional to the particle radius with a given filled-angle β.

$$F_r \propto \sigma R \sin\beta\left[-\pi\cos\left(\theta_p + \beta\right) + K\left(\beta\right)\left(1 - \cos\beta\right)\right] \qquad (14)$$

where $K(\beta) = kR$ is the function of the filled-angle β. From Eq. (14), F_r increases with the particle radius for a certain filled-angle. Therefore, the liquid will be promoted to rise under vertical capillary force with a larger particle.

However, the vertical capillary force reduces along with the particle radius decrease. The appearance of liquid lifting is conditional for the micron scale particle. The vertical capillary force should be larger than zero for lifting the solvent liquid. Then, the critical radius for zero-F_r is

derived as

$$R_{crit} = \frac{-\sin\left(\theta_p + \beta\right) + \cos\left(\theta_p + \beta\right)\sqrt{\left(\frac{\pi \sin\beta}{1-\cos\beta} + \frac{1}{\cos\left(\theta_p + \beta\right)}\right)^2 - 1}}{2k\sin\beta} \quad (15)$$

Considering the situation in this work, the void between particles and substrate is filled with solvent liquid. The critical radius at this situation is corresponding to the radius of the appearance of liquid lifting from the substrate, which can be represented as following with $\beta = \pi/2$,

$$R_l = \frac{\sigma}{\Delta p}\left[\cos\theta_p + \sin\theta_p\sqrt{\left(\pi - 1/\sin\theta_p\right)^2 - 1}\right] \quad (16)$$

The particles used in this paper are hydrophilic particles with contact angles less than 90 ^{o}. The relationship between the nondimensional radius $R_l\Delta p/\sigma$ and the contact angle is shown in Supplementary Fig. 27. As can be seen that, the critical lifting radius R_l is inversely related to the contact angle when the contact angles are less than 27.8^{o}. Within this range, a larger particle radius is needed to lift the liquid for the more hydrophilic particle. For this reason, the liquid will not be lifted for some hydrophilic particles that the size is less than R_l. Then the signal of CV molecules will is observed on the substrate. Therefore, the size of the particle selected as an aggregate medium should be larger than R_l.

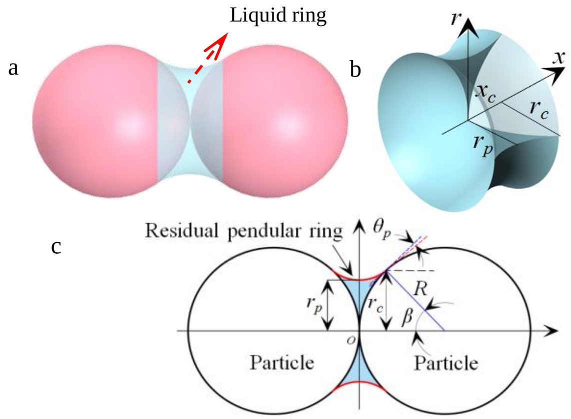

Supplementary Fig. 26 | The model of residual pendular liquid ring between spherical particles.

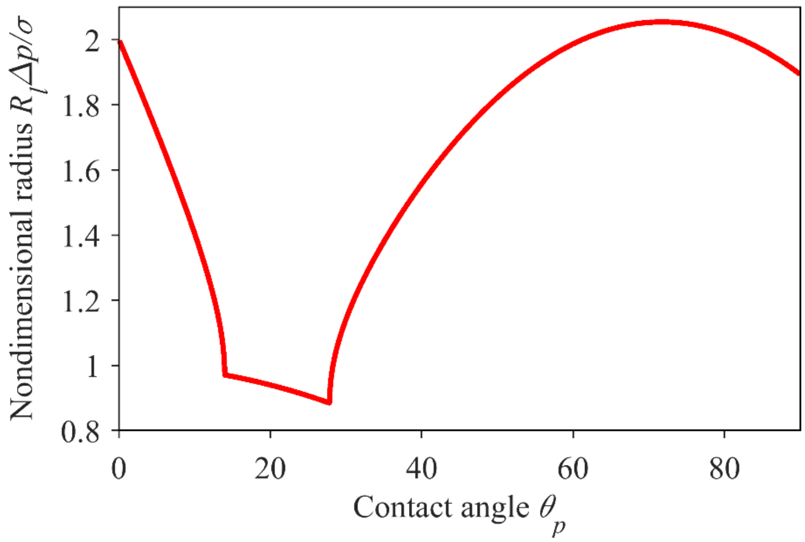

Supplementary Fig. 27 | The relationship between nondimensional radius and contact angle for the liquid lifting of Eqn. (16).

Supplementary References

1. Orr, F. M., Scriven, L. E. & Rivas, A. P. Pendular rings between solids - meniscus properties and capillary force. *Journal of Fluid Mechanics* **67**, 723-742 (1975).

2. Yamamoto, Y., Ito, T., Wakimoto, T. & Katoh, K. Numerical simulations of spontaneous capillary rises with very low capillary numbers using a front-tracking method combined with generalized Navier boundary condition. *International Journal of Multiphase Flow* **51**, 22-32 (2013).

3. Yamamoto, Y. *et al.* Numerical analysis of contact line dynamics passing over a single wettable defect on a wall. *Physics of Fluids* **28**, 082109 (2016).
