# Supplementary material for: Buoyant particulate strategy for few-to-single particle-based plasmonic enhanced nanosensors
Source: Nat Commun. 2020 May 25;11:2603. doi: 10.1038/s41467-020-16329-y (PMC7248072; doi:10.1038/s41467-020-16329-y)
Supplement: Supplementary file 3 — Description of Additional Supplementary Files [file 41467_2020_16329_MOESM3_ESM.pdf]

Description of Additional Supplementary Files

File Name: Supplementary Movie 1
Descriptions: The evaporation processes of suspended hollow silica-coated Au shell particles within a droplet.

File Name: Supplementary Movie 2
Descriptions: The aggregation and enrichment processes of suspended hollow silica-coated Au shell particles on a slippery surface. The number of buoyant-particulates is more than 10.

File Name: Supplementary Movie 3
Descriptions: The aggregation and enrichment processes of suspended hollow silica-coated Au shell particles on a slippery surface. The number of buoyant-particulates is 2.

File Name: Supplementary Movie 4
Descriptions: The in-situ moving operations of the aggregated particles on a slippery surface.
